# Prevalence, Severity, and Correlates of Anaemia in Pregnancy among Antenatal Attendees in Warri, South-Southern Nigeria: A Cross-Sectional and Hospital-Based Study

**DOI:** 10.1155/2020/1915231

**Published:** 2020-05-08

**Authors:** Victor Omote, Henry Awele Ukwamedua, Nathaniel Bini, Emmanuel Kashibu, Joel Rimamnde Ubandoma, Akafa Ranyang

**Affiliations:** ^1^Department of Laboratory Services, Central Hospital Warri, Warri, Nigeria; ^2^Department of Medical Laboratory Science, Faculty of Basic Medical Sciences, Ambrose Alli University, Ekpoma, Nigeria; ^3^Department of Medical Laboratory Science, Faculty of Health Sciences, Taraba State University, Jalingo, Nigeria; ^4^Department of Public Health, Faculty of Health Sciences, Taraba State University, Jalingo, Nigeria; ^5^Department of Obstetrics and Gynecology, Taraba State Specialist Hospital, Jalingo, Nigeria

## Abstract

**Introduction:**

Anaemia in pregnancy affects about half of all pregnant women globally and constitutes an important reproductive health issue. The World Health Organization estimates that the prevalence of anaemia in pregnancy varies from 53.8% to 90.2% in developing countries and 8.3% to 23% in developed countries. Anaemia in pregnancy is common in developing countries and prevalence statistics required for its effective management and control is not adequately available in Nigeria. Thus, this study seeks to provide prevalence statistics of anaemia in pregnancy for the study region and its severity and highlight some possible correlates.

**Methods:**

A total of 218 pregnant women were recruited from the antenatal clinic of Central Hospital Warri using simple random technique after approval from the institutional review board and consent from the participants. Data on sociodemographics, economic status, and clinical history were collected using a pretested structured interviewer's questionnaire. Participant's haematocrit levels were estimated using standard laboratory techniques and anaemia was diagnosed using WHO-recommended cutoff.

**Results:**

The overall prevalence of anaemia was 37.6%. This prevalence dropped to 10.6% when a cutoff of less than 30% haematocrit was used. There was a direct relationship between haematocrit values and the participants' age while mild anaemia accounted for the bulk (72%) of the anaemic cases. Participants younger than 20 years of age gave the highest age-based prevalence while parity-based prevalence was even among subgroups. Participants without formal education and those who were unemployed accounted for the highest prevalence in their different categories.

**Conclusion:**

Based on the findings from our study, anaemia in pregnancy is still of primary public health concern if WHO cutoff is used for the diagnosis. However, the reduction observed when less than 30% haematocrit was used tags our study zone to be of moderate severity. Although all variables lacked statistical significance, younger age, no formal education, and unemployment were highlighted to be predisposing factors.

## 1. Introduction

Anaemia is defined as a decrease in red cell haemoglobin concentration in relation to age, sex, and geographical specifications. It is termed as one of the most common nutritional deficiency globally and affects more than 1.6 billion people worldwide [1]. Of the estimated 1.6 billion people, 56 million are pregnant women [2].

Anaemia in pregnancy affects about half of all pregnant women globally and constitutes an important global/reproductive health issue [3]. The World Health Organization estimates that the prevalence of anaemia in pregnancy varies from 53.8% to 90.2% in developing countries and 8.3% to 23% in developed countries [4].

According to the Centre for Disease Control and Prevention, anaemia in pregnancy is defined as haemoglobin levels of less than 11 g/L (haematocrit less than 33%) [5]. Anaemia in pregnancy ranges from mild (10.0–10.9 g/L or haematocrit of 30–32.9%), moderate (7–9.9 g/L or 21–29.9%) to severe (less than 7 g/L or less than 21%) [6].

Maternal anaemia is multifactorial with regard to possible causes. It could be physiological (haemdilution or/and increase in nutritional demands from the growing foetus), nutritional (deficiency of iron, vitamins, and other micronutrients), genetic (haemoglobinopathies), and infection-induced (malaria, intestinal infestation, tuberculosis, and HIV).

The morbidity and mortality rate of the mother/child pair resulting from maternal anaemia is alarming. According to “The Saving Mothers Report (2010–2013),” 40% of maternal deaths in South Africa were associated with anaemia [7]. Foetal consequences associated with anaemia in pregnancy include but are not limited to still-births, low weight babies, intrauterine growth restriction, and neonatal sepsis.

Anaemia in pregnancy is common in developing countries and prevalence statistics required for its effective management and control is not adequately available in Nigeria. Thus, this study seeks to provide prevalence statistics of anaemia in pregnancy for the study region, assay for its severity, and highlight some possible correlates.

## 2. Materials and Methods

### 2.1. Study Region, Design, and Population

This study was conducted at the Central Hospital Warri. Central Hospital Warri is a 350-bed-secondary health-care facility located in the oil-rich city of Warri, Delta State, Nigeria. It serves as a referral centre for some parts of Delta, Edo, and Bayelsa State. Warri is the most populated city in Delta state and serves as home to most ethnic nationalities in Nigeria. The study was institution-based, descriptive, and cross-sectional in design and it included 218 pregnant women who attended the antenatal clinic of Central Hospital Warri, from May to August 2019.

### 2.2. Sampling Technique

All pregnant women visiting the study site during the study duration who were not critically ill were eligible for the study. A total of 218 pregnant women were recruited using a simple random sampling technique based on the calculated sample size using the formula as proposed by Araoye [8].

(1) $$\begin{array}{c} N=\frac{Z^{2}\times P\times q}{d^{2}}, \end{array}$$

where *Z* is the critical value and in a two-tailed test, *Z* = 1.96. *P* is the estimated prevalence of 20.7% [9], *q* is the probability which is 1 − *P*, while *d* is the absolute sampling error that can be tolerated. In this study, it will be 5%. Thus,

(2) $$\begin{array}{c} N=\frac{{\left(1.96\right)}^{2}\times 0.20\times \left(1-0.20\right)}{{\left(0.05\right)}^{2}},\\ N=\frac{3.8416\times 0.20\times 0.80}{0.0025},\\ N=\frac{0.6147}{0.0025},\\ N=246. \end{array}$$

However, we had a response rate of 88.7% as only 218 participants willingly responded.

### 2.3. Ethical Clearance

The study design and methodology were reviewed by the ethical committee of Central Hospital Warri and approval was granted. Written consent was obtained from consenting participants after the consent form was properly explained to them. Confidentiality was ensured as identifiers were not collected during data collection and access to the data collected was restricted to the research team.

### 2.4. Data Collection and Laboratory Analysis

A pretested structured interviewer's questionnaire was used to collect data on demographics and socioeconomic status by trained research assistants. HIV status was extracted from their case records.

About three millilitres (3 mL) of venous blood was collected from each consenting participant following standard aseptic procedures. The blood sample was placed into a labelled EDTA container. PCV was estimated using a standard laboratory technique and procedure. Anaemia was diagnosed using the WHO-recommended cutoff of less than 33%. Participants were categorized into a subgroup of anaemia severity using the WHO cutoffs for mild, moderate, and severe anaemia [6].

### 2.5. Data Management and Analysis

Data was collected and entered into the database on a weekly basis after cleaning. Confidentiality of data collected was ensured as identifiers were not included and data access was strictly restricted to the research team. At the end of the study, data was subject to descriptive and inferential statistical analysis using Statistical Package for Social Science (SPSS) (Version 22.0) (IBM) statistical software. Continuous variables were expressed in mean and standard deviation while categorical variables were summarized as percentages. Chi-square and Fisher's exact test were used to assay for the association between study variables. *P* values of ≤0.05 were considered to be significant.

## 3. Results

A total of 218 participants with a mean age of 30.65 ± 5.52 years were involved in the study. The youngest was 12 years old while the oldest was 47 years old. Almost half of the participants, 104 (47.7%), were within 21–30 years. Multiparous participants were 106 (48.6%) while self-employed respondents were 148 (67.9%). Women with tertiary education were 104 (47.7%) while urban residents and participants with gravidity of 2–4 accounted for 194 (89.0%) and 123 (56.4%), respectively. Characteristics of the studied population are detailed in Table 1.

We recorded a mean ± SD PCV of 33.12% ± 3.17. The mode PCV was 34.14% while the maximum and minimum PCV values were 52% and 21%, respectively. An overall prevalence of 37.6% (82/218) was recorded in this study (Table 2).

**Figure 1:**
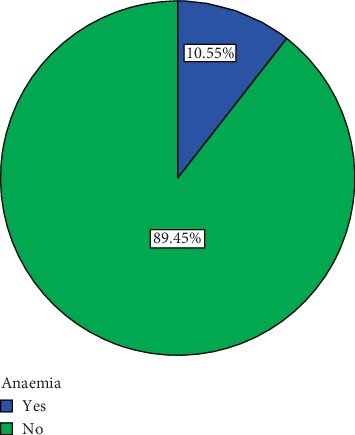
Overall prevalence of anaemia when less than 30% was used as a cutoff.

When a haematocrit cutoff of less than 30% was used, the prevalence dropped from 37.6% to 10.6%. Details are shown in Figure 1.

The majority of the anaemic cases were mild 72% (59/82). There was no case of severe anaemia as moderate anaemia accounted for the remaining cases (Table 3).

There were varying degrees of the prevalence of anaemia in pregnancy among the different study variables and all variables lacked statistical association with anaemia in pregnancy. Participants with age lesser than 20 years had the highest age-based prevalence; it was an even distribution based on parity, women without formal education gave the highest educational status-based prevalence while unemployed women topped the occupation-based prevalence (Table 4).

**Figure 2:**
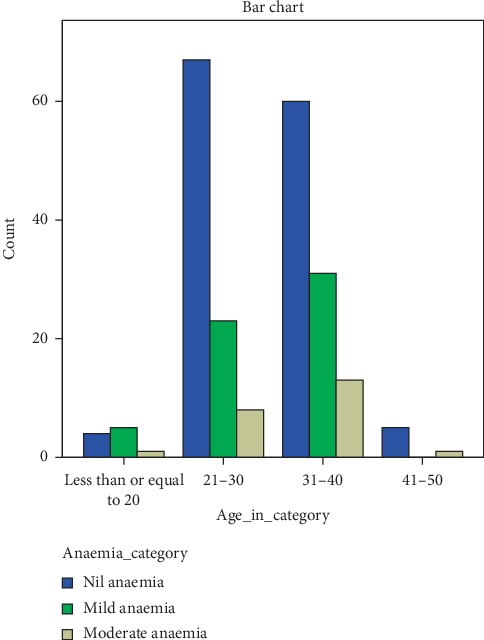
Age-based distribution of anaemia according to its severity.

**Figure 3:**
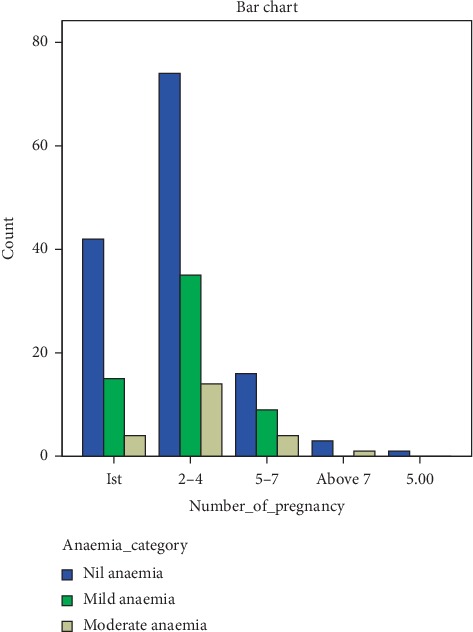
Anaemia severity based on the number of pregnancies.

Anaemia severity gave varying distributions among age-groups and number of pregnancies (Figures 2 and 3). Women within the age group of 31–40 gave the highest number of mild anaemic participants while women within the age group of 41–50 had no case of mild anaemia but one case of moderate anaemia. Similar trend was observed for the number of pregnancies. Women with 2–4 gave the highest number of participants with mild anaemia.

## 4. Discussion

This study was carried out to evaluate the prevalence of anaemia in pregnancy among antenatal attendees at the Central Hospital Warri, South-Southern Nigeria, and to identify possible correlates.

We recorded a prevalence of 37.6% (82/218) which is compared favourably with the reports of Ikeanyi and Ibrahim [10] (32.2%) for Benin city, 35.2% recorded for Lagos by Anorlu et al. [11], and 40% reported by Dim and Onah [12] for Enugu state in Southern Nigeria. Our finding is also consistent with reports from Ethiopia [13] and India [14] but is lower than several other Nigerian reports [3, 15, 16]. Reports from an urban city in India [17] and Tanzania [18] were also higher than our report. Sociocultural, economic, and geographical variations among regions and countries may be responsible for the variance in prevalence of anaemia in pregnant women. Morbidity/clinical conditions such as HIV, malaria, parasitaemia, sickle cell anaemia, and thalassemia which happens to be prevalent in certain regions or/and countries may also affect the prevalence pattern. Study design and target population may also be a contributing factor as institution-based study tends to give figure quite different from community-based surveys. Lastly, the variation in haemoglobin or PCV values for the diagnosis of anaemia in pregnancy is another factor. While WHO recommends HB less than 11 g/dl or PCV less than 33%, some studies [19, 20] used PCV of less than 30%.

When a haematocrit cutoff of less than 30% was used for the diagnosis of maternal anaemia, the prevalence dropped from 37.6% to 10.6% (Figure 1). The debate of an appropriate cutoff for the diagnosis of anaemia in pregnancy for the study region has been an intense one for over some years now. Anaemia generally depends on several factors among which geographical location is very vital. The mean PCV from our study was 33.12% while the standard deviation was ±3.17. Using our data as a point of reference, it will be more appropriate to use PCV less than 30% for the diagnosis of anaemia for the study region. Olubukola et al. [20] reported 30–33% as the commonest range of PCV for women who go through pregnancy without any apparent ill effects to themselves or their offsprings.

**Figure 4:**
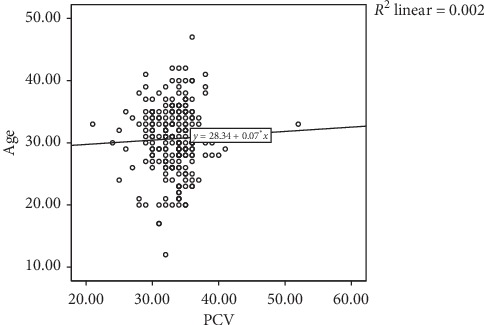
Correlation between maternal age and PCV.

PCV values increased as age increased (Figure 4). This finding is in consonance with the report of Buhari et al. [21] but in contrast to the report of Gwarzo and Ugwa [22].

The majority of the anaemic cases were mild (72%) while the remainders were moderate cases (28%). Our report is in agreement with the record of Onoh et al. [3] for Abakaliki Southeast Nigeria and Aimakhu and Oluyemi report for Southwest Nigeria [23] but in disagreement with others reports where mild and moderate anaemia has similar proportion or/and cases of severe anaemia recorded [14, 24–26]. The nature of our study area may be responsible for the pattern of anaemia severity observed. Warri is a megacity and happens to be the economic hub of the oil-rich Delta state. Thus, the sociocultural and economic status of the participants irrespective of their social class would have supported social and dietary habits that discourage anaemia. Being a megacity, there is improved access to healthcare and reduced risk of clinical conditions that encourages anaemia (all participants were HIV negative).

Participants within the less than 20-year age group gave the highest age-based prevalence of 60%. This report is in agreement with several other reports [25, 27–29] but disagrees with the reports of Udukwu and Dienye [30] and Sholeye et al. [31]. The high prevalence associated with younger age may be attributed to the lack of awareness, poor knowledge of antenatal services, failure to book for ANC early, and mental/psychological stress associated with teenage pregnancy since the bulk of them were unwanted, unplanned for, and out of wedlock. Societal stigmatization may also cause depression which was reported to have significant association with maternal anaemia [14].

Several reports have associated increased parity with increased risk of anaemia in pregnancy [32, 33]. Our findings are in contrast as anaemia prevalence was even among the different parity subgroups. It is worth noting that the reports cited above are for rural settings. These reports associated maternal bleeding during pregnancy with iron store depletion as factors responsible for the direct relationship between parity and anaemia in pregnancy. However, early registration for antenatal services, adequate spacing of pregnancy, and access to quality and timely health-care services which are peculiar to our study participants and region have also been documented to reduce the risk for anaemia in pregnancy.

Participants without formal education gave a 50% prevalence which happens to be the highest educational status-based prevalence. However, similar prevalence was reported for participants of the other educational status-based subgroups. Our finding is in agreement with Chowdhury et al. [34] and Stephen et al. [35]. Educational status influences the maximization of antenatal services and the consumption of nutritious diets. These may be responsible for the effect of educational status on maternal anaemia.

Participants that were unemployed had the highest occupation-based prevalence of 42.9%. This record is consistent with the findings of Sumitra and Kumari [36] who reported that women who were completely dependent on others account for the highest occupational status-based prevalence. Occupational status has a direct relationship with the level of income. Thus, women who are unemployed will have little or no income to purchase the right quality and quantity of food required to prevent anaemia.

## 5. Conclusion

Based on the findings from our study, anaemia in pregnancy is still of primary public health concern if WHO cutoff is used for the diagnosis. However, the reduction observed when less than 30% haematocrit was used tags our study zone to be of moderate severity. Although all variables lacked statistical significance, younger age, no formal education, and unemployment were highlighted to be predisposing factors.

## Figures and Tables

**Table 1:** Characteristics of the studied population.

Characteristics	Frequency	Percentage
Age (years)		
11–20	10	4.6
21–30	98	45.0
31–40	104	47.7
Above 40	6	2.8

Parity		
Nulliparous or primiparous	98	45.0
Multiparous	106	48.6
Grand multiparous	14	6.4

Employment status		
Student	11	5.0
Professionals	45	20.6
Self-employed	148	67.9
Nonemployed	14	6.4

Educational status		
None	2	0.9
Primary	12	5.5
Secondary	100	45.9
Tertiary	104	47.7

Location of residence		
Urban	194	89.0
Semiurban	21	11.0
Rural	0	0.0

Gravidity		
First	61	27.9
2^nd^–4^th^	123	56.4
5^th^–7^th^	29	13.4
Above 7^th^	5	2.3

**Table 2:** Overall prevalence of anaemia in pregnancy.

Anaemic status	Frequency	Percentage
Anaemic	82	37.6
Normal	136	62.4

**Table 3:** Distribution of anaemia in pregnancy according to severity.

Category of anaemia	Frequency	Percentage
Severe	0	0
Moderate	23	28
Mild	59	72
Total	82	100

**Table 4:** Distribution of anaemia in pregnancy among the study variables.

Variables	Frequency	Anaemic (*n*) (%)	Nonanaemic (*n*) (%)	*P* value
Age (years)				0.126
<20	10	6 (10.0)	4 (40)	
21–30	98	31 (31.6)	67 (68.4)	
31–40	104	44 (42.3)	60 (57.7)	
Above 40	6	1 (16.7)	5 (83.3)	

Parity				0.989
Null/primi	98	37 (37.8)	61 (62.2)	
Multi	106	40 (37.7)	66 (62.3)	
Grand multi	14	5 (35.7)	9 (64.3)	

Employment status				0.948
Nonemployed	14	6 (42.9)	8 (57.1)	
Student	11	4 (36.4)	7 (63.6)	
Self-employed	148	54 (36.5)	94 (63.5)	
Professional	45	18 (40.0)	27 (60.0)	

Educational status				0.972
None	2	1 (50.0)	1 (50.0)	
Primary	12	4 (33.3)	8 (66.7)	
Secondary	100	38 (38.0)	62 (62.0)	
Tertiary	104	39 (37.5)	65 (62.5)	

Location of residence				1.000
Urban	194	72 (37.6)	121 (62.4)	
Semiurban	24	9 (37.5)	15 (62.5)	
Rural	0	0 (0.0)	0 (0.0)	

Gravidity				0.581
First	61	19 (31.1)	42 (68.9)	
2^nd^–4^th^	123	49 (39.8)	72 (60.2)	
5^th^–7^th^	29	13 (44.8)	16 (55.2)	
Above 7^th^	5	1 (20.0)	4 (80.0)	

## Data Availability

The data used to support the findings of this study are available from the corresponding author upon request.
